# The impact of co-infections on fish: a review

**DOI:** 10.1186/s13567-016-0383-4

**Published:** 2016-10-04

**Authors:** Mohamed H. Kotob, Simon Menanteau-Ledouble, Gokhlesh Kumar, Mahmoud Abdelzaher, Mansour El-Matbouli

**Affiliations:** 1 Clinical Division of Fish Medicine, Department for Farm Animals and Veterinary Public Health, University of Veterinary Medicine, Vienna, Austria; 2 Department of Pathology, Faculty of Veterinary Medicine, Assiut University, Asyut, Egypt

## Abstract

Co-infections are very common in nature and occur when hosts are infected by two or more different pathogens either by simultaneous or secondary infections so that two or more infectious agents are active together in the same host. Co-infections have a fundamental effect and can alter the course and the severity of different fish diseases. However, co-infection effect has still received limited scrutiny in aquatic animals like fish and available data on this subject is still scarce. The susceptibility of fish to different pathogens could be changed during mixed infections causing the appearance of sudden fish outbreaks. In this review, we focus on the synergistic and antagonistic interactions occurring during co-infections by homologous or heterologous pathogens. We present a concise summary about the present knowledge regarding co-infections in fish. More research is needed to better understand the immune response of fish during mixed infections as these could have an important impact on the development of new strategies for disease control programs and vaccination in fish.

## Introduction

The subject of co-infections of aquatic animals by different pathogens has received little attention even though such infections are common in nature. Co-infections are defined by infection of the host by two or more genetically different pathogens where each pathogen has pathogenic effects and causes harm to the host in coincidence with other pathogens [1, 2]. Several other terms are used sometime to describe co-infections and include polymicrobial diseases, complicated infections, concurrent infections, mixed infections, multiple infections, dual infections, secondary infections and super infections [2]. Many researchers have concentrated only on single infections, classifying the other agent as opportunistic and mostly ignoring it so in this review article we will focus on distinguishing the infections caused by more than one organism. During episodes of co-infection, interactions between the infectious agents yield to varied outcomes: the load of one or both pathogens may be increased, one or both may be suppressed or one may be increased and the other suppressed [1]. The natural environments in which animals live are varied and harbor a variety of heterogeneous micro-organisms including parasitic and non-parasitic species and co-infection are a frequent occurrence. There is therefore, a considerable necessity to investigate the interactions occurring between these species during mixed infections and the deleterious effects of multi-infections on fish disease pathogenesis, prognosis, and treatment [3–5].

During co-infections, pathogens can compete with each other for resources or target sites inside the same host. Alternatively, sometimes one pathogen can alter the immune response of the host against the subsequent infections by other pathogens either by suppressing or priming the immune system [6, 7]. This can result in a change of the host susceptibility to infection and affect the host-pathogen dynamics, infection biology, disease severity, duration of infection and host pathology [7, 8]. Therefore, the interactions between co-occurring pathogens can be either synergistic or antagonistic [1, 9]. Synergistic effects can occur when the first pathogen induces immunosuppression in the host and hinders the immune response against subsequent infections, leading to an increase in the severity of the infections and mortality rates [7, 9]. Antagonistic effects, however, can result from competition of direct pathogens for nutrients and places and limit the population size of the infectious agents and, in some cases, alter the site of infection [10]. In other cases, the antagonistic effects happen when the first pathogen triggers and modulates the host immune response and hinders the second pathogen [11].

In humans, several publications have described the effect of one pathogen on the abundance of other pathogens co-infecting the same host and the intensity of infection estimated through measures of viral load, parasitic egg counts, antibody reaction and immune response, bacterial burdens in tissues and/or the host survival rate and recovery time [4]. Immunosuppressed people infected with human immunodeficiency virus (HIV-1) have shown an increased susceptibility to secondary infections such as tuberculosis, which promotes HIV-1 replication and increases the viral load [12]. Similarly, the susceptibility of HIV-1 infected persons to secondary infection with malaria has been shown to be increased six times [13].

Chronic helminth infections, particularly in humans, produce a strong T helper 2 (Th2) and regulatory immune response. This influences the immune response to other unrelated pathogens during mixed infections, for example reducing the inflammatory response, as well as the efficacy of disease vaccines [14, 15].

In the aquatic environment, fish are commonly exposed to heterogeneous infectious macro or micro-organisms. However, little is known about how the presence of one pathogen can affect the load of other pathogens and how the host mortality rate will be changed during co-infection in comparison with single infection [9]. Because of how frequent co-infections can be and because of the potentially important impact that co-infections can have on the development of a disease, it is important to understand how defensive immunity to a specific pathogen can occur in the host infected with multiple pathogens. Studying different co-infection models is central to the development of new effective vaccination and disease control strategies [6]. In the present article, we review recent studies on co-infections of fish by homologous and heterologous pathogens. The impact of these co-infections on the susceptibility of the fish, the course and severity of the infection and the interactions between different pathogens are also reviewed in different co-infection models. Tables 1 and 2 summarize the different interactions occurring between different homologous and heterologous pathogens in fish during co-infections.

**Table 1:** **Summary of different interactions occurring during co-infections by different homologous pathogens in different fish species**

Host species	First pathogen	Second pathogen	Type of interaction during co-infection	References
**Bacterial co-infections**				
Atlantic salmon, *Salmo salar*	*Aliivibrio wodanis*	*Moritella viscosa*	Antagonistic	[24, 25]
Vietnamese catfish, *Pangasianodon hypophthalmus*	*Edwardsiella ictaluri*	*Aeromonas hydrophila*	Synergistic	[17]
Thailand striped catfish, *Pangasianodon hypophthalmus*	*E. ictaluri*	*Flavobacterium columnare*	Synergistic	[18]
Chinook salmon, *Oncorhynchus tshawytscha*	*Renibacterium salmoninarum*	*A. hydrophila*	Synergistic	[19]
**Viral co-infections**				
Grouper fin cells, GF-1	Snakehead retrovirus	Grouper nervous necrosis virus	Synergistic	[27]
Channel catfish ovary and brown bullhead cells	Channel catfish reovirus	Ictalurid herpesvirus 1	Antagonistic	[29]
Rainbow trout, *Oncorhynchus mykiss*	Infectious hematopoietic necrosis	Viral hemorrhagic septicemia virus	Antagonistic	[30]
Rainbow trout, *Oncorhynchus mykiss*	Infectious pancreatic necrosis virus	Infectious hematopoietic necrosis	Antagonistic	[36]
Chinook Salmon Embryo Cells, CHSE-214	Salmonid Alphavirus	Infectious pancreatic necrosis virus	Antagonistic	[41]
Japanese Flounder, *Paralichthys olivaceus*	Aquabirnavirus	Viral hemorrhagic septicemia virus	Antagonistic	[42]
Atlantic salmon, *Salmo salar*	Infectious pancreatic necrosis virus	Infectious salmon anaemia virus	Antagonistic	[81]
Olive flounder, *Paralichthys olivaceus*	Marine birnavirus	Nervous necrosis virus,VHSV, Lymphocystis disease virus	Synergistic	[83]
Japanese flounder, *Paralichthys olivaceus*	Aquabirnavirus	Viral hemorrhagic septicemia virus	Antagonistic	[85]
**Parasitic co-infections**				
Farmed brown trout, *Salmo trutta*	*Tetracapsuloides bryosalmonae* (Myxozoa)	*Chloromyxum schurovi* (Myxozoa)	Antagonistic	[48, 49]
Wild brown trout, *Salmo trutta*	*T. bryosalmonae*	*Raphidascaris acus* (Nematode)	Synergistic	[50]
Farmed lumpfish, *Cyclopterus lumpus*	*Nucleospora cyclopteri* (Microsporidia)	*Kudoa islandica* (Myxozoa)	Synergistic	[51]
Atlantic salmon, *Salmo salar*	*Caligus rogercresseyi* (Sea louse)	*Neoparamoeba perurans* (Protozoa)	Synergistic	[52]
Atlantic salmon, *Salmo salar*	*Lepeophtheirus salmonis* (Fish louse)	*N.perurans* (Protozoa)	Synergistic	[53]

**Table 2:** **Summary of different interactions occurring during co-infections by different heterologous pathogens in different fish species**

Host species	First pathogen	Second pathogen	Type of interaction during co-infection	References
**Parasitic and bacterial co-infections**				
Rainbow trout, *Oncorhynchus mykiss*	*Myxobolus cerebralis* (Myxozoa)	*Yersinia ruckeri*	Synergistic	[75]
Nile tilapia, *Oreochromis niloticus*	*Gyrodactylus niloticus* (Helminth)	*Streptococcus iniae*	Synergistic	[59]
Goldfish, *Carassius auratus*	*Dactylogyrus intermedius* (Helminth)	*F. columnare*	Synergistic	[60]
Rainbow trout, *Oncorhynchus mykiss*	*Argulus coregoni* (Fish louse)	*F. columnare*	Synergistic	[57]
Channel catfish, *Ictalurus punctatus*	*Ichthyophthirius multifiliis* (protozoa)	*E. ictaluri*	Synergistic	[65]
Nile tilapia, *Oreochromis niloticus*	*I. multifiliis*	*S. iniae*	Synergistic	[70]
Channel catfish, *Ictalurus punctatus*	*I. multifiliis*	*A. hydrophila*	Synergistic	[72]
Atlantic salmon, *Salmo salar*	*Piscirickettsia salmonis*	*C. rogercresseyi*	Synergistic	[62]
Channel catfish, *Ictalurus punctatus*	*E. ictaluri*	*I. multifiliis*	Synergistic	[64]
Channel catfish, *Ictalurus punctatus*	*S. iniae* or *S. agalactiae*	*Trichdina* sp.	Synergistic	[71]
**Parasitic and viral co-infections**				
Whiting, *Merlangius merlangus euxinus*	Viral hemorrhagic septicemia virus	*Trichdina* sp.	Synergistic	[76]
**Bacterial and viral co-infections**				
Atlantic salmon, *Salmo salar*	Infectious pancreatic necrosis virus	*Vibrio salmonicida*	Synergistic	[81]
Grouper, *Epinephelus* sp.	Infectious pancreatic necrosis virus	*Vibrio carchariae*	Synergistic	[82]
Olive flounder, *Paralichthys olivaceus*	Marine birnavirus	*S. iniae*, *Vibrio spp*.	Synergistic	[83]
Olive flounder, *Paralichthys olivaceus*	Marine birnavirus	*Vibrio harveyi* or *E. tarda*	Synergistic	[84]
Japanese flounder, *Paralichthys olivaceus*	Aquabirnavirus	*E. tarda* or *S. iniae*	Synergistic	[85]
Rainbow trout, *Oncorhynchus mykiss*	*Flavobacterium psychrophilum*	Infectious pancreatic necrosis virus	Synergistic	[86]
**Fungal and bacterial co-infections**				
Nile tilapia, *Oreochromis niloticus*	*Fusarium oxysporum*	*A. hydrophila*	Synergistic	[87]
Discus fish, *Symphysodon*	*Fusarium solani*, *F. oxysporum* or *F. moniliform*	*A. hydrophila*	Synergistic	[88]

## Co-infections with homologous pathogens

### Bacterial co-infections

The subject of bacterial co-infections in fish is one that has yet to receive the scrutiny it deserves and includes dual, triple or multiple bacterial infections. It has been reported that artificial infection of channel catfish, *Ictalurus punctatus* by the enterobacterium *Edwardsiella ictaluri* elicits a bacteraemia with motile aeromonad species, *Aeromonas hydrophila* [16]. This was confirmed later by Crumlish et al. [17] who repeated these results with Vietnamese catfish, *Pangasianodon hypophthalmus*. These authors, however, showed that the reverse is not true and that artificial infection with *A. hydrophila* does not result in shedding of *E. ictaluri* [17]. Moreover, artificial co-infection challenge of Vietnamese catfish with both bacteria using an immersion route caused higher cumulative mortalities (95%) in the co-infected group and (80%) in *E. ictaluri* only infected fish when compared to the very low mortalities (10%) in the fish exposed to *A. hydrophila* alone [17]. Based on these results, the authors suggest that while *E. ictaluri* acts like a primary pathogen, the role of *A. hydrophila* is more opportunistic [17].

Naturally, concurrent infection of *E. ictaluri* and *Flavobacterium columnare* in striped catfish, *Pangasianodon hypophthalmus* in Thailand has also been reported [18]. Dong et al. [18] experimentally challenged the striped catfish juveniles with single and both bacteria using the immersion (i.m) and injection (i.p) routes and the results showed high cumulative mortality in co-infected fish in both i.m. and i.p. routes when compared to single infection of *E. ictaluri* or *F. columnare* and the co-infected fish showed the clinical signs of both diseases. The results obtained by Crumlish et al. [17] and Dong et al. [18] mimicked the natural outbreaks of the disease in striped catfish farms in Vietnam and Thailand.

In addition to artificial challenges, comparable results were observed in chinook salmon, *Oncorhynchus tshawytscha* where *A. hydrophila* was found associated in a higher number than predicted by chance alone in fish infected with *Renibacterium salmoninarum* [19]. Because of *R. salmoninarum* immunosuppressive qualities [20], in this case also, the authors suggest that facultatively pathogenic motile *Aeromonas* spp. act as an opportunistic pathogen and interact synergistically with *R*. *salmoninarum* [19]. In wild brown trout, *Salmo trutta* Schmidt-Posthaus et al. [21] have reported the concomitant presence of two distinct species of chlamydial bacteria (*Candidatus Piscichlamydia salmonis* and *Candidatus Clavochlamydia salmonicola*) in gill samples causing epitheliocystis of the gill lamellae. The possible interactions between these two pathogens, however, have yet to be investigated.

Finally, the “Winter Ulcer Syndrome” is a syndrome associated with skin ulcers that occurs in marine water at low temperature [22]. Two bacterial species, *Moritella viscosa* and *Aliivibrio wodanis* are often isolated together or separately from the infected fish. However, *Moritella viscosa* is the main causative agent of the disease [22, 23]. Notably, despite being cytopathogenic in vitro, *A. wodanis* appears limited in its virulence while artificial infection with *M. viscosa* induces severe clinical signs of the disease and infection with *A. wodanis* does not [24]. Moreover, co-infection with both *M. viscosa* and *A. wodanis* does not increase the mortality rate of the fish compared to infection with *M. viscosa* alone [24]. In fact, infection with *A. wodanis* appears to reduce the virulence of *M. viscosa* as prior infection with *A. wodanis* reduces the mortalities in subsequent infection with *M. viscosa* [24]. The reason for this phenomenon might be linked to the ability of *A. wodanis* to alter the gene expression profile of *M. viscosa*, likely through competition for the same niche and nutrients including the sequestration of iron through siderophore mediated interspecies competition as well as the inhibition of *M. viscosa* growth through the secretion of inhibitory effectors like bacteriocins [24, 25].

### Viral co-infections

Concomitant infections involving two or more viral pathogens have also been reported. For example Kibenge et al. [26] reported the detection of both infectious salmon anemia (ISA) and an unknown togavirus-like virus in Atlantic salmon *Salmo salar*. Interestingly, the togavirus was isolated and used in a challenge in the absence of ISAV and found to be avirulent, suggesting that it played no role in the disease etiology [26]. On the contrary, co-infection with snakehead retrovirus (SnRV) was reported to increase the infection titer and the cytopathic effects (CPE) of Grouper nervous necrosis virus (GNNV), a member of the Nodaviridae, in vitro in Grouper fin cell line (GF-1) [27]. Notably, this effect was not found in other cell lines and this constitutes an example of interference of the life cycle of fish nodavirus with fish retrovirus.

More interesting is the phenomenon of viral interference which is defined by the ability of one virus to interfere with the replication of another virus that has been reported between several aquatic viruses. Viral inference occurs as a result of several mechanisms including the inhibition by one virus of the multiplication of a second virus or interference with the entry of the virus through down regulation of viral receptors or direct competition between viruses for a common receptor [28, 29]. Moreover, infection with the first virus can also inhibit or alter some functions in the host cell that are required by the second invading virus. Finally, first viral infection can induce interferons or anti-viral factors that inhibit the replications of the second virus [29]. An example of viral interference occurs during co-infection with channel catfish reovirus (CRV) and Ictalurid herpesvirus 1(CCV), where CRV was found to reduce both viral titers and CPE of CCV in vitro [29]. This interference induced by CRV was considerable when the cell culture was first infected with CRV then co-infected with CCV after 16 h but not when infection was performed simultaneously [29]. Moreover, it was found that infectious hematopoietic necrosis virus (IHNV) infections were hindered in the presence of viral hemorrhagic septicemia virus (VHSV) infections in rainbow trout, *Oncorhynchus mykiss* and resulted in a more restricted distribution of IHNV among the fish internal organs [30]. The authors suggest that this interference and antagonistic effect might be due to competition for the same receptors on the surface of the cells, as antibody interference suggests the virus uses similar receptors, at least in the brain [31].

Hedrick et al. [32] also mentioned another example of viral interference occurring during co-infection by avirulent cutthroat trout virus (CTV) and IHNV and showed that prior infection of rainbow trout with CTV decreased the mortality associated with later infection by IHNV. Likewise LaPatra et al. [33] found that initial exposure to avirulent chum salmon reovirus then co-challenged with IHNV 8 weeks later resulted in an increased survival rate in rainbow trout.

However, the best studied example of viral interference in fish is probably the interactions between infectious pancreatic necrosis virus (IPNV) and IHNV. For example, the first report of IHNV in Spain was a case of dual infection alongside IPNV [34] and Alonso et al. [35] have shown that such dual infection reduces the yield of IHNV, while it has no effect on IPNV. Furthermore, the same authors later reported that such dual infection also reduces the presence of both viruses in fish leukocytes [36]. The mechanisms through which this interference occurs is still to be fully understood, however, it has been shown that interferon response is induced alongside the Mx protein [37–39], an antiviral protein that has been shown to be induced by interferon [40]. Notably, it was also shown that interferon activity is effective against IHNV but has no effect on IPNV [39], which correlates to the pattern observed during IPNV-IHNV dual infections where IHNV is hindered by the presence of IPNV.

Another example of viral interference occurred in vitro in chinook salmon embryo cells (CHSE-214) co-infected by salmonid alphavirus (SAV), and IPNV [41]. The results showed that SAV inhibits the growth and replication of IPNV to some extent. However, the opposite does not occur and this inhibition is explained by up-regulation of IFN-mediated antiviral activity and Mx expression induced by SAV infection but not by IPNV infection [41].

In Japanese Flounder, *Paralichthys olivaceus* dual infection initiated by aquabirnavirus (ABV) followed by a challenge with VHSV at 3, 7, 14 and 21 days post ABV infections suggests that the primary ABV infection provided a non-specific protection against the secondary VHSV infection. This protection started at day 3 and continued to day 14 then disappeared at day 2 post ABV exposure. The cumulative mortalities were decreased up to day 14 then increased to 90% at day 21 [42]. The authors explained this antagonistic effect and the non-specific protection against VHSV as a result of ABV induced synthesis of a potent interferon like substance with antiviral activity against VHSV [42].

### Parasitic co-infections

Parasites often exist in a dynamic equilibrium with their hosts and changes in the environment can alter the parasite/host equilibrium causing outbreaks of disease. Parasites can cause mechanical damage such as proliferation and fusion of gill lamellae and tissue replacement by the occupying parasite, physiological damage including cell proliferation, immunomodulation, change in the fish body condition or negative behavioral responses and/or affecting the reproductive capacity of fish [43–46]. Co-infections by multiple parasites have a great influence on the host–parasite ecology [47]. In farmed brown trout, mixed infections with five myxozoan species (*Tetracapsuloides bryosalmonae*, *Sphaerospora truttae*, *Chloromyxum schurovi*, *Chloromyxum truttae* and *Myxobolus* species) were observed in the samples collected from farms in central Scotland. Examined kidney samples revealed mixed infection with three myxozoan species: *T. bryosalmonae*, *S. truttae* and *C. schurovi* [48]. Infection with *T. bryosalmonae* evoked some degree of immunity against *C. schurovi* and conversely. Peeler et al. [49] mentioned the presence of a strong negative association between *T. bryosalmonae* and *C. schurovi* which was particularly apparent in the kidney. This organ acts as the target site for both parasites, and the infection by one parasite might decrease the probability of infection by the other through competition on the same target organ, however, this interaction should be explored more experimentally.

*Tetracapsuloides bryosalmonae*, a malacosporean parasite, has been responsible for proliferative kidney disease in wild brown trout and was also associated in concurrent infection with the nematodes *Raphidascaris acus*. The process of recovery from PKD was mainly influenced by the presence or absence of the nematode larvae, where brown trout without *R. acus* regenerated renal morphology completely while concurrently infected brown trout showed chronic renal lesions and incomplete translocation of *T. bryosalmonae* from the renal interstitium into the tubular lumen [50].

A case study of co-infection by *Nucleospora cyclopteri* (Microsporidia) and *Kudoa islandica* (Myxozoa) in farmed lumpfish, *Cyclopterus lumpus* L. has been reported, and the mortality rates were 65% in farmed lumpfish. Kidney, spleen and liver showed severe necrotic changes with the presence of intracellular *N. cyclopteri* in the affected tissues and *Kudoa* spores were diagnosed in the skeletal muscle, without any inflammatory response [51].

In Chile, high mortalities were reported in Atlantic salmon farms following co-infection by *Caligus rogercresseyi* and *Neoparamoeba perurans*, the causative agent of amoebic gill disease (AGD). *C. rogercresseyi* was shown to play a vital role in the transmission of *N. perurans* resulting in several outbreaks [52]. Similarly, *Lepeophtheirus salmonis*, another salmon louse similar to *C. rogercresseyi* was also found to play a similar role as a vector in the transmission of *N. perurans* and influenced the epizootiology of the disease in Atlantic salmon and increased mortalities in Atlantic salmon farms in the USA [53].

## Co-infections with heterologous pathogens

### Parasitic and bacterial co-infections

Parasitic infections increase the risk of secondary bacterial diseases and can act as a vehicle to transmit bacterial pathogens [48]. This synergistic interaction was demonstrated by many experimental studies [55–57], which showed increased mortality rates in parasitized/bacteria co-infected fish. This synergistic effect has been explained as a result of the stress caused by parasites reducing the resistance of fish to other secondary bacterial infections [58] as well as the damaging effects caused by the parasite that provided the invading of bacteria with a route of entry. In some instances, the parasites harbor the bacteria and deliver it to their host while feeding [58]. More attention should be directed toward prevention of parasitic infection in fish to reduce fish mortality due to secondary bacterial infection.

In the intensive aquaculture of Nile tilapia, *Oreochromis niloticus* mixed infections are more likely to occur and have been associated with fish losses [59]. However, most research has focused on a single parasite or a single bacterial agent. A concurrent experimental infection model of Nile tilapia was studied by Xu et al. [59] and fish were infested with *Gyrodactylus niloticus* (a monogenean helminthic ectoparasite) then challenged with a pathogenic bacteria, *Streptococcus iniae*. The results of this study showed higher mortality in a co-infected group during the first 2 weeks after exposure (42.2%) when compared to the *S. iniae* only infected group (6.7%) and no mortalities were recorded in *G. niloticus* only infected fish. Xu et al. [59] assumed that this ectoparasite provides a portal of entry for invasive bacteria through mechanical damage of the fish epithelium. Moreover, viable *S. iniae* was isolated from *G. niloticus* that acted as a mechanical vector for the bacterium [59].

Cusack and Cone [54] observed the presence of bacterial colonies on the surface of *Gyrodactylus* by scanning electron microscopy, although the precise role of these bacteria was not clear and it was not certain whether the bacteria were pathogenic to fish or not. *Dactylogyrus intermedius*, a monogenean, was also reported to increase the susceptibility of gold fish, *Carassius auratus,* to the bacterium *F. columnare,* the aetiological agent of columnaris disease, resulting in higher mortality and increasing the bacterial loads in fish tissues when compared to non-parasitized fish [60]. *D. intermedius* enhanced the bacterial invasion after induction of host immune suppression and down regulation of immune genes like TGF-β and complement 3 in gills and kidneys and thus modulate the host immune response [60].

Lhorente et al. [61] studied co-infections in Atlantic salmon experimentally by using intracellular bacteria, *Piscirickettsia salmonis* as a primary pathogen and the sea louse, *C. Rogercresseyi* agent was added 4 days after bacterial exposure at high and low doses as a secondary co-infection. In the two treatments of co-infected groups, the mortality reached up to 100% after 53 days in comparison to 46% in the single infection. This synergistic interaction was explained by the sea louse reducing the resistance of Atlantic salmon to *P. salmonis*. The authors also suggest that *C. rogercresseyi* directly damages the skin which facilitates the bacteria to invade the skin resulting in higher mortalities [62]. Similarly, in rainbow trout fish lice, *Argulus coregoni* an ectoparasite increased the susceptibility of fish to *F. columnare* and the cumulative mortality was significantly higher in the co-infected group when compared to the single infected group. Furthermore, the onset of disease and mortalities occurred earlier [57].

*Ichthyophthirius multifiliis* is a ciliated ectoparasitic fish protozoan, responsible for considerable losses in fresh water fish worldwide. It can increase bacterial invasion and fish mortality by damaging the epithelium of the gills and skin [63]. Shoemaker et al. [64] explored the effect of *I. multifiliis* parasitism on survival, hematology and bacterial burden of channel catfish exposed 1 day before to *E. ictaluri*, the causative agent of enteric septicemia of catfish. Higher bacterial load in different organs with higher mortalities were detected in the co-infected group (71.1%) when compared to single infected groups.

Xu et al. [65] designed another experiment using the same pathogens, *I. multifiliis* and *E. Ictaluri,* in channel catfish. The initial infection was performed using *I. multifiliis* then 5 days later, *E. ictaluri* was added as a concurrent infection. The results were similar to the previous trial with increased mortality rates and higher bacterial burdens in the internal organs.

In another condition, channel catfish were co-infected by *I. multifiliis* and fluorescent *E. ictaluri* at different doses and sampled at different times [66]. Hundred percent of tomonts were shown to carry the fluorescent bacteria. *E. ictaluri* survived and could replicate inside the tomonts, resulting in higher cumulative mortalities in infected fish [66]. The surface of *I. multifiliis* theronts contains carbohydrates like d-galactose, d-mannose, d-glucose, and N-acetylgalactosamine [67] and *E. ictaluri* has the ability to bind and attach to these carbohydrate molecules [68]. Therefore, the binding of *E. ictaluri* to *I. multifiliis* during co-infection occurs as a result of the interaction between the *E. ictaluri* lectin-like receptors and *I. multifiliis* surface d-galactose or d-mannose. This binding does not affect the replication of *I. multifiliis*, movement and its attachment to the host [66].

**Figure 1:**
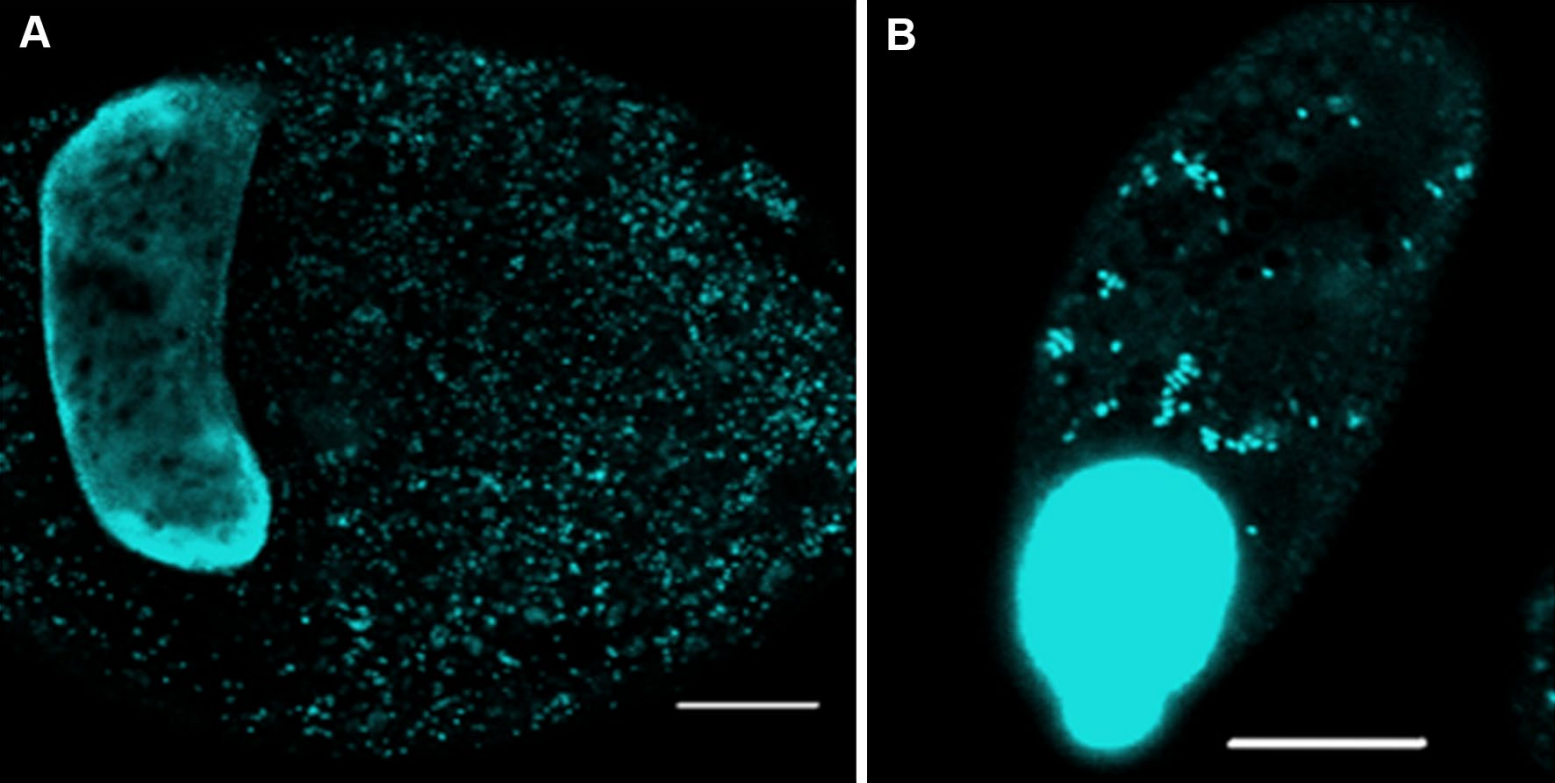
**DAPI-stained confocal images of an** ***I. multifiliis tomont and theront.*** **A** Macronucleus and endosymbiotic bacteria (blue) in G13 tomont. Bar = 100 µm. **B** Micronucleus merged with macronucleus and endosymbiotic bacteria (blue) in G13 theront. Scale bar = 10 µm. (Image from Sun et al. [69] with permission).

**Figure 2:**
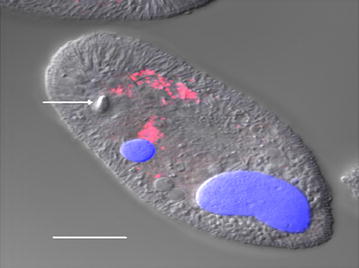
**FISH image of an** ***I. multifiliis*** **G5 theront labeled with bacterial probe EUB338 and counterstained with DAPI.** FISH and DAPI merged confocal image showing endosymbiotic bacteria labeled with probe (red), DAPI-stained micro and macronucleus (blue) and the organelle of Lieberkühn (arrow). Scale bar = 10 µm. (Image from Sun et al. [69] with permission).

16S rRNA gene sequences from three bacterial classes, *Alphaproteobacteria (Rickettsiales)*, *Sphingobacteria*, and *Flavobacterium columnare* were identified in the PCR product of two isolates of *I. multifiliis* [69]. DAPI (4′,6-diamidino-2-phenylindole) showed the presence of these bacteria in the cytoplasm of trophont and theronts as shown in Figure 1 [69]. Fluorescent in situ hybridization (FISH) showed only *Rickettsiales* and *Sphingobacteriales* classes as endosymbiotic bacteria in the cytoplasm of the parasite but *Flavobacterium columnare* was not detected suggesting that it may adhere to *I. multifiliis* through the cilia as shown in Figure 2 [69].

In Nile tilapia, Xu et al. [70] established a co-infection model with *I. multifiliis* and *S. iniae* and found a strong relation between the parasite load, its developmental size and fish mortality. Increasing the time interval between exposures to both pathogens during co-infection allowed more time for *I. multifiliis* to produce large, well developed trophonts that caused more damage to the epithelium of fish and increased the bacterial invasion. This resulted in higher mortalities than when the fish were only exposed to young small trophonts [70].

In channel catfish the susceptibility to *S. iniae* or *S. agalactiae* was greatly increased after concurrent parasitism with *Trichdina* sp. with mortalities reaching 100%. This synergistic interaction between the external parasite and bacteria were explained to be a result of the damaging effect of *Trichdina* to the skin of fish which enhanced the invasion of *S. iniae* or *S. agalactiae* after immersion exposure [71].

Another co-infection trial between protozoa and bacteria was conducted by Xu et al. [72] to determine whether co-infection of *I. multifiliis* parasitized channel catfish with *A. hydrophila* increased fish mortality rates or not. The results confirmed that the *I. multifiliis* parasitized catfish showed significantly higher mortality (80%) after being exposed to *A. hydrophila* and had a higher load of *A. hydrophila* in the internal organs. *I. multifiliis* infection significantly increases the cortisol level in rainbow trout thus leading to immune suppression of fish and this synergistic effect [73, 74].

A mixed infection between *Myxobolus cerebralis*, the causative agent of whirling disease and *Yersinia ruckeri*, the causative agent of enteric red mouth disease was also reported by Densmore et al. [75]. Chronically, *M. cerebralis* infected rainbow trout after 12 months post exposure were bath challenged with *Y. ruckeri*. The total mortality rates in *M. Cerebralis*–*Y. ruckeri* co-infected group was higher than the non *M. cerebralis* infected group and the onset of mortality occurred much faster. These results were likely due to the immunomodulatory effects of *M. cerebralis* via suppression of lymphocyte blastogenesis and lowered proliferative lymphocyte responses to four mitogens. This resulted in greater bactericidal activity and could affect secondary infection by *Y. ruckeri* [75].

### Parasitic and viral co-infections

The co-occurrence of VHSV and *Trichodina* ectoparasite was reported in whiting (*Merlangius merlangus euxinus*) collected from the black sea area. In a field study, a relationship was demonstrated between virus loads and the presence of ectoparasites as the burdens of *Trichodina* spp. were higher in VHSV infected whiting than non VHSV infected fish. These data indicate that the load of these ectoparasites, possibly in conjunction with other factors such as spawning or water temperatures, has a significant effect on the occurrence of VHSV in whiting [76].

Nylund et al. [77] explored the role of salmon lice (*C. elongates* and *L. salmonis*) as a vector for the transmission of ISAV, including through the occurrence of skin damage and immunosuppression, resulting in epizootic outbreaks and mortalities. Valdes-Donoso et al. [78] mentioned that ISAV outbreaks that occurred in southern Chile between 2007 and 2009 resulted from co-infection of Atlantic salmon by ISAV and sea lice.

Finally, high mortalities (100%) occurred in American bullfrog larva in Florida, following co-infection by alveolate parasite infections and frog virus 3-like ranavirus [79]. Early investigations suggest that the alveolate parasite is the main pathogen in these outbreaks and that co-infection with the virus is secondary. However, this secondary infection increases the severity of the outbreak and the rate of mortality. The details of the interactions between the parasite and the virus, however, are still unclear [79].

### Bacterial and viral co-infections

Several outbreaks in newly cultured sparid fish species were recorded and isolation and characterization of causative agents revealed the presence of both bacteria and virus in affected fish. The isolated bacteria were identified as *Vibrio* spp. and *Photobacterium damselae* subsp. *damselae* while the presence of viral nervous necrosis virus (VNNV) and VHSV were also confirmed in the same infected fish samples alongside the bacteria. These results suggest that co-infection of fish with different bacteria and viruses could occur and result in these outbreaks [80].

The influence of bacterial and viral co-infection was studied in Atlantic salmon. Fish were infected first with IPNV before being challenged with either ISAV or *V. salmonicida*. The cumulative mortality was observed to be higher in IPNV–*V. salmonicida* co-infected group than in IPNV-free fish challenged with *V. salmonicida* alone. The onsets of mortalities started earlier in the co-infected group (3–4 days) in comparison with fish infected with *V. salmonicida* only (8 days) confirming the synergistic interaction between both pathogens [79]. On the contrary, secondary exposure of acute IPNV infected Atlantic salmon with ISAV resulted in lower mortalities than fish infected with ISAV only, illustrating an antagonistic effect of IPNV against ISAV which provided some protection against the development of ISAV through the production of interferon (IFN) or IFN-like agents in response to acute IPNV infection [81].

Lee et al. [82] investigated the effect of a double challenge with IPNV and *V. carchariae* in grouper (*Epinephelus* sp.), using an initial challenge with IPNV followed 2 weeks later with a secondary infection with *V. carchariae*. No mortalities after IPNV exposure were recorded while secondary exposure with *V. carchariae* caused 100% mortalities.

Mass mortalities were reported in cultured olive flounder, *Paralichthys olivaceus* in Korea. Samples of these infected fish were examined for bacterial and viral diseases using PCR and sequence analysis and revealed the presence of different strains of marine aquabirnavirus (MABV). MABV has been associated with low mortalities in fish. However, it can also be found in association with other bacteria (*S. iniae*, *Vibrio* spp., *V. harveyi* and *E. tarda*) or other viruses (VNNV, VHSV, lymphocystis disease virus), in which case it causes higher mortalities [83, 84]. In Japanese flounder, Pakingking et al. [85] mentioned different interactions between ABV and other pathogens such as VHSV, *E. tarda* or *S. iniae*. The interaction was synergistic between ABV and *E. tarda* or *S. iniae* and enhanced the secondary bacterial infection and resulted in higher mortalities (84%) compared to other single infected groups. On the contrary, the interaction was antagonistic between ABV and VHS, resulting in lower mortalities compared with fish infected with VHSV alone.

**Figure 3:**
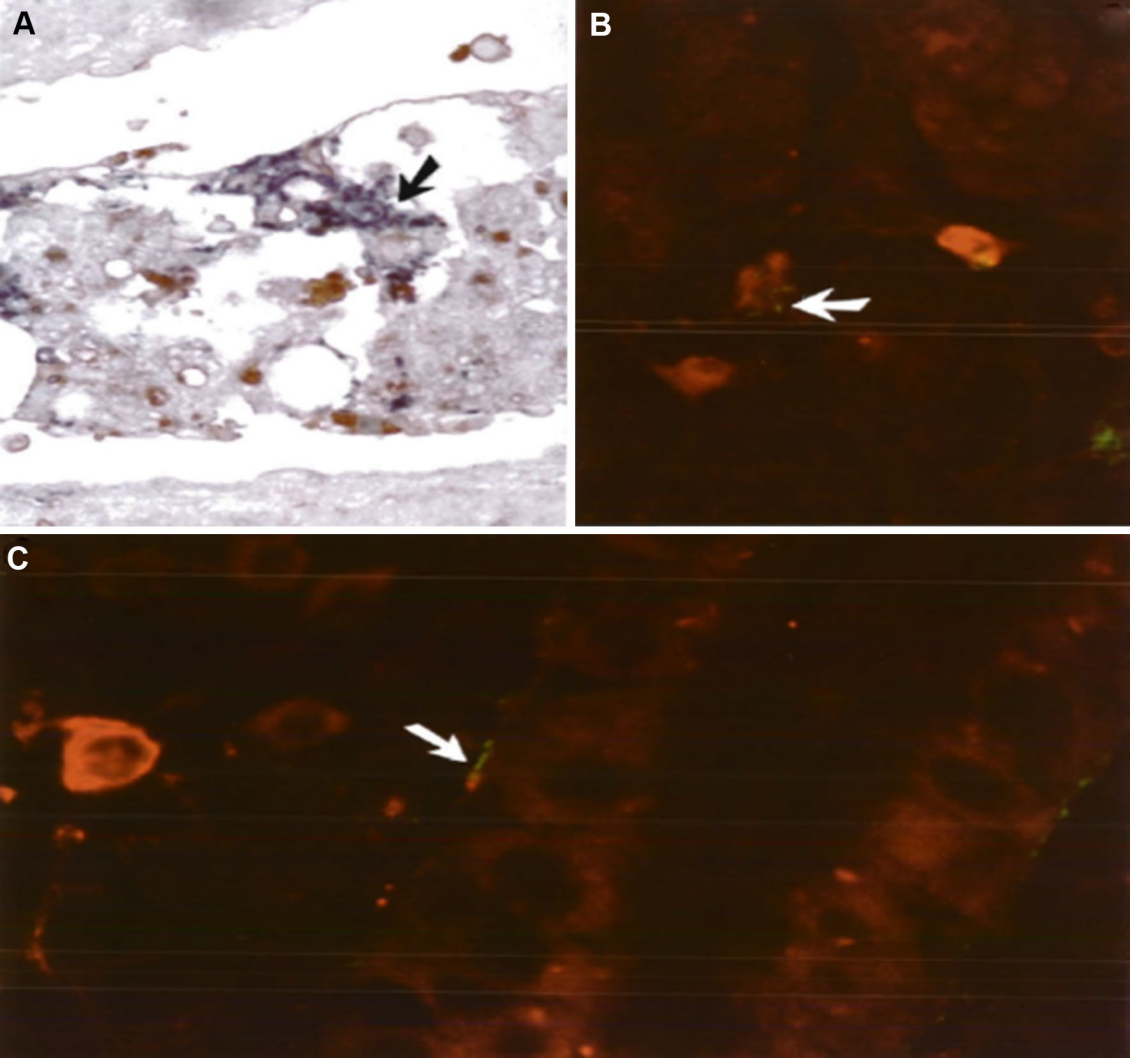
**Immunohistochemial tissue sections from rainbow trout fry concurrently infected with** ***F. psychrophilum*** **and infectious pancreatic necrosis virus (IPNV). A** Immunohistochemia image for exocrine pancreas shows blue reaction for *F. psychrophilum* and red-brown for IPN virus. Note single bacteria (arrow), without counterstaining, X530. **B** Immunofluorescent section from head kidney shows red fluorescence for IPN virus in the cytoplasm of interstitial cells and the presence of bacteria either alone or as a group at the lower right with green fluorescence, X660. **C** Higher power magnification for immunofluorescent mid kidney shows IPN virus in the cytoplasm of the interstitial cell. Endothelial cells lining the tubulus show positive staining for both IPN virus and *F. psychrophilum* possibly in the same cell (arrow), X833. (Image from Evensen and Lorenzen [86] with permission).

In Denmark, several outbreaks due to the rainbow trout fry syndrome (RTFS), which is caused by the Gram-negative bacterium, *F. psychrophilum*, have occurred, resulting in high mortalities in rainbow trout fry. IPNV was also isolated from infected fry as a concomitant infection and it has been difficult to determine which pathogen was the primary cause of mortality in such outbreaks or to recognize this synergistic interaction between those two pathogens [86]. Immunohistochemistry revealed the presence of IPNV in the necrosed cells of the exocrine pancreas and *F. psychrophilum* in the interstitial tissues adjacent to the infected pancreatic islets (as shown in Figures 3A), however, both pathogens were detected in the same endothelial cell of the head and middle kidney (as shown in Figures 3B and C) [86].

### Fungal and bacterial co-infections

Fungal infections have been reported in farmed and marine fish species and the first case of fungal-bacterial co-infection in fish has been recently reported by Cutuli et al. [87], where *Fusarium oxysporum* was diagnosed in the skin of Nile tilapia and co-occurred with *A. hydrophila*. The histopathological results showed severe congestion of the hepatopancreas with necrotic foci in the hepatic tissue infiltrated with large numbers of neutrophil cells. The fungal agent caused tissue damage, therefore facilitating the invasion of *A. hydrophila*, increasing the mortality of the fish [87].

In Egypt, discus fish (*Symphysodon*) collected from a local fish farm after the sudden onset of mortalities with eye cloudiness, ascites, extreme body mucus and tail rot were found to harbour different kinds of fungi such as *Fusarium solani*, *F. oxysporum* and *F. moniliform*. The bacteria *A. hydrophila* was also re-isolated from 60% of the examined cases and the fish parasite, dinoflagellate *Spironucleus* spp. from 80% of infected cases. This suggests the causative agents of Discus mortalities to be a complex of several pathogens like fungi, bacteria, and parasites [88].

## Conclusions

The main aim of this review was to summarize the scant literature regarding the interactions between different pathogens during co-infections of the fish host with more than one infectious agent either by simultaneous or secondary infections. The interactions can be either synergistic or antagonistic and might result in the enhancement or inhibition of one or both pathogens, increasing or decreasing the severity of the disease. Such interactions can have an important impact on the development and severity of the diseases and should be considered during the planning of therapy and vaccination. It is evident that more research is needed in the future to improve our understanding on the interactions between fish pathogens and how they interact with the immune response of the fish host. This will deepen our understanding of the disease process and pathogenesis and will prove useful for disease management.

## Abbreviations

*A. hydrophila*: *Aeromonas hydrophila*
*E. ictaluri*: *Edwardsiella ictaluri*
*F. columnare*: *Flavobacterium columnare*
*I. multifiliis*: *Ichthyophthirius multifiliis*
IHNV: infectious hematopoietic necrosis virus
VHSV: viral hemorrhagic septicemia virus
IPNV: infectious pancreatic necrosis virus
ISAV: infectious salmon anaemia virus
VNNV: viral nervous necrosis virus
*C. rogercresseyi*: *Caligus rogercresseyi*
DAPI: 4′,6-diamidino-2-phenylindole
FISH: fluorescence in situ hybridization
CPE: Cytopathic effects
