# Erratum to: The impact of co-infections on fish: a review

**DOI:** 10.1186/s13567-017-0432-7

**Published:** 2017-04-19

**Authors:** Mohamed H. Kotob, Simon Menanteau-Ledouble, Gokhlesh Kumar, Mahmoud Abdelzaher, Mansour El-Matbouli

**Affiliations:** 1 Clinical Division of Fish Medicine, Department for Farm Animals and Veterinary Public Health, University of Veterinary Medicine, Vienna, Austria; 2 Department of Pathology, Faculty of Veterinary Medicine, Assiut University, Asyut, Egypt

## Erratum to: Vet Res (2016) 47:98 DOI 10.1186/s13567-016-0383-4

Following publication of this article [1], an error was brought to our attention in the below paragraph.

In Chile, high mortalities were reported in Atlantic salmon farms following co-infection by *Caligus rogercresseyi* and *Neoparamoeba perurans*, the causative agent of amoebic gill disease (AGD). *C. rogercresseyi* was shown to play a vital role in the transmission of *N. perurans* resulting in several outbreaks [52]. Similarly, *Lepeophtheirus salmonis*, another salmon louse similar to *C. rogercresseyi* was also found to play a similar role as a vector in the transmission of *N. perurans* and influenced the epizootiology of the disease in Atlantic salmon and increased mortalities in Atlantic salmon farms in the USA [53].

The correct version should be read as follows:

“In Chile, the first outbreak of amoebic gill disease (a condition caused by *Neoparamoeba perurans*) in Atlantic salmon occurred in fish heavily infected with the sea louse *Caligus rogercresseyi* [52]. This suggested that *C. rogercresseyi* might have contributed to the outbreak. Alternatively, it is also possible that both parasites took advantage of a third factor, for example the poor overall health of the fish or the unusually high salinity of the water at the time of the outbreak as both parasites are known to thrive under such high salinity conditions [52].

DNA from *N. perurans* was identified in samples of the sea louse *Lepeophtheirus salmonis* from a location in the Puget Sound with a prior history of AGD [53]. This suggested that *L. salmonis* harboured the amoeba, possibly on its carapace and that it might play a role in its dispersion.

Interestingly, despite sea lice’s absence from Tasmania, AGD is a recurring problem in this region which signifies that the copepod is not essential for AGD outbreak [53].”
